# The symptom network of oral health conditions in older populations with oral frailty: a cross-sectional study

**DOI:** 10.1186/s12903-025-05795-9

**Published:** 2025-04-01

**Authors:** Zhang Chengrui, Xing Ying, Luan Wei, Chen Bin

**Affiliations:** 1 Shanghai Jiao Tong University School of Nursing, Shanghai, China; 2 Shuguang Hospital Affiliated to Shanghai University of TCM, Shanghai, China; 3 Medical Administration Division, Minhang District Health Commission, Shanghai, China

**Keywords:** Oral frailty, Network analysis, Geriatrics

## Abstract

**Background:**

Population aging is increasing globally, with oral frailty affecting 24% of the older population. Previous studies have demonstrated the relationship between oral frailty and poor oral health symptoms but the interactions and core symptoms among these oral symptoms remain vague.

**Objective:**

To clarify the relationship between oral frailty and oral symptoms, explore the intrinsic connections between these symptoms, and identify core symptoms to provide more effective management and treatment strategies for oral frailty and related health issues.

**Methods:**

The study conducted a cross-sectional study from January 2024 to April 2024, included 547 participants using a convenient sampling method. The study adopted the Geriatric Self-Efficacy Scale for Oral Health (GSEOH), Oral Frailty Index-8, FRAIL scale, and oral symptoms, including the simplified oral hygiene index score (OHI-S), plaque index, periodontal pocket depth and gingival index, teeth numbers and tooth mobility index. T-test, Chi-Square test, Mann–Whitney test and binary logistic regression were used to explore the relationship between oral symptoms and oral frailty, and symptom network analysis and subgroup network analysis were used to explore the intrinsic connections between oral symptoms and identify core symptoms.

**Results:**

The study found that while teeth number and GSEOH were significantly associated with oral frailty (teeth number: β = -0.262, *P* = 0.013) (GSEOH: β = -0.056, *P* = 0.000), the centrality of teeth number and GSEOH was the lowest compared to other oral symptoms. oral hygiene status (CI-S and plaque index), and gingival index were the core symptoms in older adults, and gingival index was the strongest bridge node.

**Conclusion:**

The study explored the relationship between oral symptoms and oral frailty using network analysis. The study recommended that communities implement routine oral functional assessments to increase the denture restoration rate and identify oral frailty at an early stage, as well as educational and promotional programs aimed at maintaining oral hygiene and function. Future research should further analyze the causal relationships between oral symptoms, particularly periodontal pocket depth, oral frailty, and overall frailty.

**Supplementary Information:**

The online version contains supplementary material available at 10.1186/s12903-025-05795-9.

## Introduction

Population ageing is becoming a major medical and social issue worldwide. 16% of the world's population will be over the age of 65 projected by 2050. Frailty is a significant health concern in the old population. The prevalence of frailty is 20.5% among community-dwelling older adults in Asia, and prefrailty is 49.3% [1, 2]. Oral frailty is defined as the abnormal oral structure and/or decline in multi-faceted oral function, accompanied by decline in physical, cognitive and social functions [3]. Oral frailty is an important part of frailty, and is highly related to worse functional recovery, prolonged time to discharge, and increased in-hospital mortality rates [4]. The prevalence of oral frailty was 24% globally, and 45.9% in China, and oral frailty has become a serious health problem [5].

Previous studies have demonstrated the relationship between oral frailty and poor oral health symptoms, such as oral hygiene, periodontal disease, tooth loss, and the number of natural teeth. A concept analysis identified severe periodontitis as the antecedent of oral frailty [3, 6, 7]. However, the interactions among these oral symptoms remain vague and the core symptoms of oral frailty have not been elucidated.

Network analysis offers an insight into investigating the relationship between oral frailty and oral symptoms by viewing these symptoms as interdependent rather than isolated [8]. Symptom network theory posits that symptoms are connected through various mechanisms, forming complex networks where one symptom may influence the development or persistence of others [9]. This interconnectedness can lead to feedback loops and alternative remission pathways, underscoring the dynamic nature of symptom interactions [10]. The study aimed to clarify the relationship between oral frailty and oral symptoms, explore the intrinsic connections between these symptoms, and identify core symptoms to provide more effective management and treatment strategies for oral frailty and related health issues. The current hypothesis is that the core symptom of oral frailty is related to severe periodontitis.

## Method

The reporting of the study followed Strengthening the Reporting of Observational Studies in Epidemiology (STROBE) Statement guidelines.

### Study design and participants

A cross-sectional study was conducted from January 2024 to April 2024, using a convenient sampling method. Inclusion criteria: (1) age ≥ 60 years; (2) have clear consciousness and comprehension ability; (3) community resident; (4) informed consent, have signed the informed consent form and participate voluntarily. Exclusion criteria: (1) hearing impairment; (2) have difficulty in opening their mouths and unable to cooperate with the oral examination; (3) have serious organic diseases or in the terminal stage of the disease.

### Sample size and data collection

The study included a total of 35 symptoms, with a sample size 5 to 10 times the number of variables. Considering a 15% attrition rate, the sample size should be at least 525 [11]. The study collaborated with five community centers in Shanghai, where trained community nurses conducted questionnaire surveys and measured oral health indicators among community older adults.

## Measures

### Oral symptoms

#### Oral hygiene status

The study used the simplified oral hygiene index score (OHI-S) and plaque index to evaluate oral hygiene status. OHIS was proposed and recommended by Greene and Vermillion in 1964 as an efficient indicator for assessing oral hygiene [12]. OHIS includes debris index score (DI-S) and calculus index score (CI-S). DI-S included 4 levels, 1 = No debris or stain present; 2 = Soft debris covering not more than one third of the tooth surface, or presence of extrinsic stains without other debris regardless of surface area covered; 3 = Soft debris covering more than one third, but not more than two thirds, of the exposed tooth surface;4 = Soft debris covering more than two thirds of the exposed tooth surface. CI-S included 4 levels, 1 = No debris or stain present; 2 = Soft debris covering not more than one third of the tooth surface, or presence of extrinsic stains without other debris regardless of surface area covered; 3 = Soft debris covering more than one third, but not more than two thirds, of the exposed tooth surface; 4 = Soft debris covering more than two thirds of the exposed tooth surface. Dental plaque can be defined as a biofilm structured in an extracellular matrix of polymers of host and microbial origin and is an important indicator of oral hygiene [13]. Plaque index included 4 levels, 1 = no visible plaque; 2 = plaque islands on the proximal surfaces; 3 = plaque islands cervical to the bracket in addition to the proximal surfaces; 4 = plaque covers > 1/3 of the surface cervical to the bracket.

#### Periodontal health status

The study used the periodontal pocket depth and gingival index. Periodontal pocket depth is a main indicator for periodontal inflammation [14]. Periodontal pocket depth was classified into 3 levels, 1 = 0–3 mm, indicating no/mild periodontitis; 2 = at least 1 pocket ≥ 4 mm and < 6 mm, indicating moderate; 3 = at least 1 pocket ≥ 6 mm, indicating severe periodontitis. Gingival Index, proposed by Loe and Silness in 1963, was designed to assess the severity and extent of gingival inflammation [15]. Gingival index includes 4 levels, 1 = healthy gums, 2 = slight color changes, light edema and no presence of bleeding on probing, 3 = edema with slight redness and bleeding on probing, 4 = severe edema, redness, the presence of ulceration and a tendency for spontaneous bleeding.

#### Teeth health status

The study used teeth numbers and tooth mobility index. Tooth mobility is a useful clinical indicator of the health of the tooth-supporting structures which can reflect tooth health [16]. Tooth mobility index included 4 levels: 1 = < 1 mm mobility; 2 = > 1 mm but not depressible; 3 = > 1 mm and depressible [17]. Teeth numbers referred to functional teeth number, including the functioning natural teeth number, the number of artificial teeth on removable dentures being worn during the oral examination, dummies on fixed partial dentures and implant-supported artificial teeth [18]. Third molars were also counted, and the teeth numbers ranged from 0 to 32.

#### Geriatric self-efficacy scale for oral health

The study used the Geriatric Self-Efficacy Scale for Oral Health (GSEOH) to assess oral health-related self-efficacy in older adults. The scale includes 3 dimensions, including oral hygiene habits (items 1–8), oral functioning (items 9–17), and dental visit (items 18–20). It utilizes a 4-point Likert scale, with 1 indicating no confidence at all and 4 indicating high level of confidence. The total score ranges from 20 to 80, with higher scores indicating higher levels of self-efficacy. The scale demonstrates good reliability, with a Cronbach's α of 0.924 [19, 20].

#### Oral frailty

The study used the Oral Frailty Index-8 (OFI-8) to evaluate oral frailty of the older adults. OFI-8 was proposed by Tomoki Tanaka to help screen older adults at risk of oral frailty in the community setting, and translated into Chinese by Chen et al. in 2023 [21, 22]. The OFI-8 comprises eight items, with a total score ranging from 0 to 11 points. Higher scores indicate poorer oral health. The OFI-8 assigns double the points to three high-priority items, reflecting their importance in assessing oral frailty. The eight items included in the OFI-8 are as follows: 1) Do you use dentures? (Yes: 2 points). 2) Have you developed any difficulties eating tough foods compared to 6 months ago? (Yes: 2 points). 3) Can you eat hard foods like squid jerky or pickled radish? (No: 1 point). 4) Do you often have a dry mouth? (Yes: 1 point). 5) Have you recently choked on your tea or soup? (Yes: 2 points). 6) How many times do you brush your teeth in a day? (< 3 times/day: 1 point). 7) Do you visit a dental clinic at least once per year? (No: 1 point). 8) Do you go out less frequently than you did last year? (Yes: 1 point).

In the Chinese version of the OFI-8, the Cronbach's α is 0.949, the split-half reliability coefficient is 0.889, and the test–retest reliability coefficient is 0.786. These results indicate that the scale demonstrates excellent internal consistency and good stability [22].

#### Frailty

The study used FRAIL scale to evaluated frailty of the older adults, which was recommended by Geriatric Medicine Branch of Chinese Medical Association [23]. The FRAIL scale includes 5 components: Fatigue, Resistance, Ambulation, Illness, and Loss of weight. Frail scale scores range from 0–5 (0 = best to 5 = worst) and represent frail (3–5), pre-frail (1–2), and robust (0) health status [24].

#### Related variables

Sociodemographic characteristics obtained: age, gender, height, weight, age, BMI, smoking, drinking, education, marital status, family income, residence, medication used number, and chronic diseases number.

### Statistical analysis

All statistical analyses were conducted using R version 1.6.4. The demographic characteristics and oral symptoms are described using frequencies, percentages, means, and standard deviations. T test, Chi-Square test, and Mann–Whitney test were used to test the difference of the older adults with and without oral frailty. Binary logistic regression analysis was performed with oral frailty as the dependent variable.

The 7 oral symptoms and oral health-related self-efficacy were included in the network analysis. In the network, each node represents one symptom. Edges in the network represented the conditional independent relationships between 2 nodes, and thicker edges indicates stronger association between the 2 nodes. The color of edges indicates the direction of the association (green edges indicate positive associations, red edges indicate negative associations). The spring layout was used to generate the undirected association networks. Symptoms with stronger and more numerous associations was placed closer to other symptoms and more centrally within the network [25, 26].

Centrality analysis was conducted to calculate three parameters: strength, betweenness, and closeness. These parameters were utilized to identify core symptoms of oral frailty from a mechanistic perspective, which also serves to validate the research hypotheses. Among these, strength centrality is considered the most important parameter. A symptom with high strength centrality is more likely to co-occur with multiple other symptoms, indicating its central role in the symptom network. Betweenness centrality reflects a symptom's influence within the network by acting as a bridge between other symptoms, while closeness centrality, defined as the inverse of the average shortest path length between a node and all other nodes, measures how closely a symptom is linked to the entire network.

Bridge analysis was also conducted to assess the bridge strength of symptoms, focusing on their role in connecting distinct symptom clusters. A symptom with high bridge strength acts as a critical connection point, facilitating interactions between two or more symptom clusters. This is particularly important for identifying symptoms that may propagate maladaptive interactions across oral symptom clusters. Such insights are invaluable for targeting key symptoms in intervention strategies and improving the management of oral frailty.

A difference test was conducted to identify significant differences in network connections and centrality measures for various variables. Bootstrapped tests compared edge weights and centrality indices in LASSO-regularized partial correlation networks, based on polychoric correlation matrices.

Bootstrapping methods were used to assess the accuracy and stability of the network with the R package bootnet. The accuracy of the estimated network connections was evaluated using 95% confidence intervals (CIs) of the edge weight values. Stability was assessed by calculating the correlation stability coefficient of the expected impact of nodes with a case-dropping subset bootstrap (1000 bootstrap samples). The correlation stability coefficient should preferably be > 0.5, but at the very least > 0.25.

A subgroup analysis of global strength using the Network Comparison Test (NCT) was performed to identify the difference in networks between older adults with and without oral frailty along with overall frailty [10, 27].

### Ethics approval and consent to participate

The study received approval from the Shuguang Hospital Affiliated to Shanghai University of TCM, Shuguang Hospital Ethics Committee (reference number: SGYYIT-2023-105) and adhered to the principles of the Declaration of Helsinki. The questionnaire's cover page outlined the study's purpose, voluntary participation, confidentiality measures, potential risks and benefits, and implied consent upon submission. All participants provided their informed consent to participate in the study.

## Result

### Characteristics of participants

This study included 547 participants in the analysis. The characteristics of the participants are shown in Table 1. The result showed that Male are significantly more likely to develop into oral frailty. Participants with oral frailty are significantly older, have more Chronic diseases and Medication used, and lower levels of education and family income. (Table 1).

**Table 1:** Characteristics of participants with and without oral frailty in Shanghai from January 2024 to April 2024 (*n* = 547)

**Characteristics**		**Oral frailty**			
		No (n (%), Mean ± SD)	Yes (n (%), Mean ± SD)	statistic	*P*
**Age**^**a**^		64.20 (8.97)	67.53(9.88)	-4.111	**0.000**
**BMI**^**a**^		24.39(5.82)	24.12(4.14)	0.641	0.522
**Gender**^**c**^	Male	79(14.44%)	115(21.02%)	5.010	**0.025**
	Female	179(32.72%)	174(31.81%)		
**Smoking**^**c**^	Yes	36(6.58%)	41(7.50%)	0.006	0.938
	No	222(40.59%)	248(45.34%)		
**Drinking**^**c**^	Yes	37(6.76%)	43(7.86%)	0.032	0.859
	No	221(40.40%)	246(44.97%)		
**Education**^**b**^	Junior high and below	126(23.03%)	177(32.36%)	3.255	**0.001**
	High school/secondary school	64(11.70%)	66(12.07%)		
	College/university and above	68(12.43%)	46(8.41%)		
**Marital Status**^**c**^	Married (including cohabiting)	235(42.96%)	259(47.35%)	0.335	0.563
	Unmarried (including divorced and bereaved)	23(4.20%)	30(5.48%)		
**Family Income**^**b**^	< 20 k	22(4.02%)	57(10.42%)	3.763	**0.000**
	20 k ~ 50 k	86(15.72%)	98(17.92%)		
	50 k ~ 80 k	76(13.89%)	80(14.63%)		
	> 80 k	74(13.53%)	54(9.87%)		
**Residence**^**c**^	Living alone	16(2.93%)	15(2.74%)	0.261	0.610
	Not living alone	242(44.24%)	274(50.09%)		
**Medication Used Number**^**a**^		2.29(1.35)	1.62(1.73)	3.256	**0.001**
**Chronic Diseases Number**^**a**^		0.90(0.89)	1.22(0.10)	4.011	**0.000**

a, t test; b, Z test; c, χ^2^ test

### Periodontal symptom prevalence and severity

The results suggest that Participants with oral frailty have significant worse situations in tooth number, tooth mobility, DI-S, CI-S, plaque index, gingival index, periodontal pocket depth, and GSEOH score. Moreover, oral frailty is significantly related to frailty. (Table 2).

**Table 2:** Periodontal symptom prevalence and severity in participants with and without oral frailty in Shanghai from January 2024 to April 2024 (*n* = 547)

**Parameters**		Oral frailty			
		No (n (%), Mean ± SD)	Yes (n (%), Mean ± SD)	Z	*P*
**Tooth Mobility**	< 1 mm mobility	225(41.13%)	228(41.68%)	-2.496	**0.013**
	> 1 mm but not depressible	28(5.12%)	57(10.42%)		
	> 1 mm and depressible	5(0.91%)	4(0.73%)		
**DI-S**	No debris or stain present	130(23.77%)	78(14.26%)	-5.955	**0.000**
	Soft debris or extrinsic stains covering ≤ 1/3 of tooth surface	111(20.29%)	163(29.80%)		
	Soft debris covering > 1/3 but ≤ 2/3 of the tooth surface	13(2.38%)	44(8.04%)		
	Soft debris covering > 2/3 of the tooth surface	4(0.73%)	4(0.73%)		
**CI-S**	No calculus present	130(23.77%)	74(13.53%)	-6.172	**0.000**
	Supragingival calculus covering ≤ 1/3 of the tooth surface	110(20.11%)	168(30.71%)		
	Supragingival calculus covering > 1/3 but ≤ 2/3 of the tooth surface, or subgingival calculus flecks around the cervical portion	15(2.74%)	42(7.68%)		
	Supragingival calculus covering > 2/3 of the tooth surface, or continuous heavy band of subgingival calculus around the cervical portion	3(0.55%)	5(0.91%)		
**Plaque Index**	No visible plaque	136(24.86%)	79(14.44%)	-6.029	**0.000**
	Plaque islands on the proximal surfaces,	106(19.38%)	173(31.63%)		
	Plaque islands cervical to the bracket in addition to the proximal surfaces	12(2.19%)	34(6.22%)		
	Plaque covering > 1/3 of the surface cervical to the bracket	4(0.73%)	3(0.55%)		
**Gingival Index**	Healthy gums	134(24.50%)	73(13.35%)	-6.373	**0.000**
	Slight color changes, mild edema, no bleeding on probing	108(19.74%)	178(32.54%)		
	Edema with slight redness and bleeding on probing	13(2.38%)	36(6.58%)		
	Severe edema, redness, ulceration, and spontaneous bleeding	3(0.55%)	2(0.37%)		
**Periodontal Pocket Depth**	≤ 3 mm	186(34.00%)	164(29.98%)	-3.929	**0.000**
	4 ~ 5 mm	52(9.51%)	78(14.26%)		
	≥ 6 mm	20(3.66%)	47(8.59%)		
**GSEOH**		65.52(12.50)	59.70(12.61)	5.408	**0.000**
**Teeth Number**		25.21(6.82)	20.69(9.24)	6.542	**0.000**
**Frailty**		2.03(0.857)	2.22(0.776)	2.73389	**0.006**

### Associated factors with oral frailty

A binary logistic regression analysis was performed with oral frailty as the dependent variable. Variable coding details are provided in the Supplementary 3.1. The results indicated that family income, teeth number, GSEOH score, and frailty were risk factors for oral frailty in older adults in the community. (Table 3).

**Table 3:** Risk factors related to oral frailty in Shanghai from January 2024 to April 2024 (*n* = 547)

**Parameters**	B	SE	Wald χ2	P	OR
Constant	1.180	1.466	0.648	0.421	3.256
Age	0.017	0.012	1.925	0.165	1.017
Gender	-0.304	0.208	2.142	0.143	0.738
Education	-0.011	0.136	0.006	0.936	0.989
Family Income	-0.262	0.106	6.121	**0.013**	0.770
Chronic Diseases	0.206	0.150	1.895	0.169	1.229
Medication Used	-0.023	0.093	0.063	0.803	0.977
Teeth Number	-0.056	0.013	17.548	**0.000**	0.946
Tooth Mobility	-0.485	0.267	3.307	0.069	0.616
DI-S	0.381	0.242	2.475	0.116	1.464
CI-S	0.073	0.284	0.066	0.797	1.076
Plaque Index	-0.055	0.289	0.037	0.848	0.946
Gingival Index	0.485	0.272	3.181	0.075	1.624
Periodontal Pocket Depth	0.023	0.164	0.020	0.887	1.024
GSEOH	-0.025	0.009	8.298	**0.004**	0.976
Fraillty	0.320	0.131	5.974	**0.015**	1.377

-2 log-likelihood = 640

Nagelkerke's R^2^ = 0.256

Hosmer–Lemeshow test *P* = 0.003

### Network structure

**Fig. 1:**
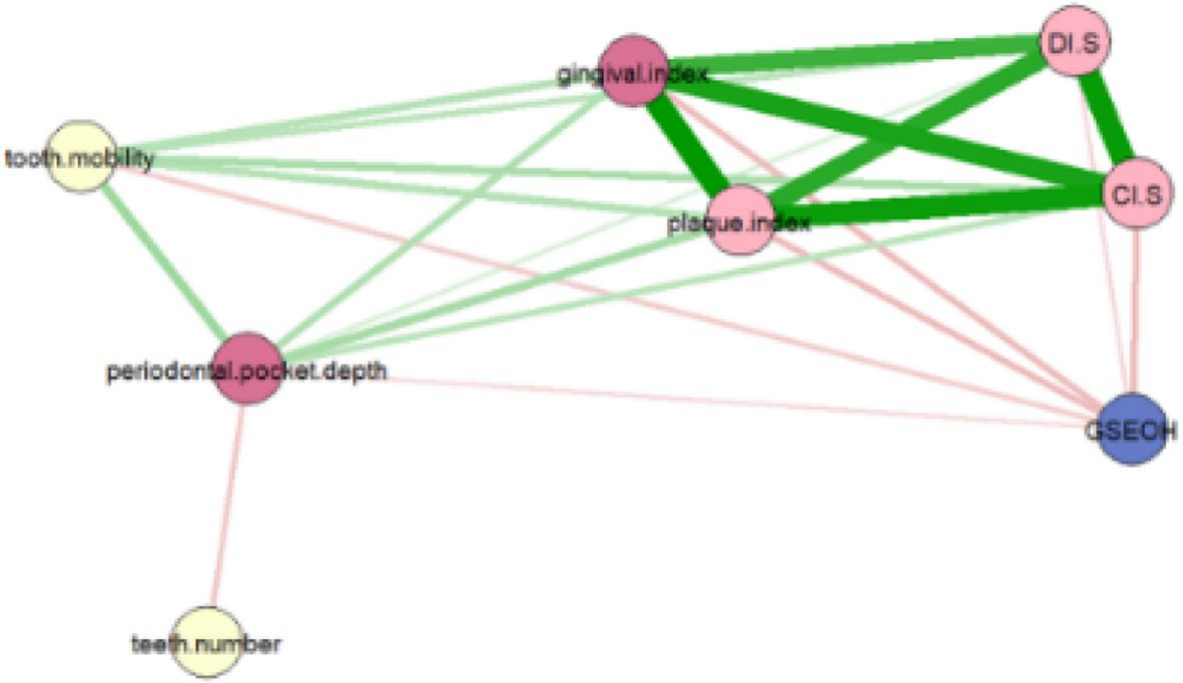
Network of oral symptoms

The network of oral symptoms was organized around the complex of DI-S, CI-S, plaque index, and gingival index, and the complex presented the strongest connection internally. Teeth numbers were situated at the periphery of the network and connected with periodontal pocket depth only. GSEOH showed negative connection with most oral symptoms except teeth number. (Fig. 1).

### Centrality, bridge centrality and difference test

#### Centrality

**Fig. 2:**
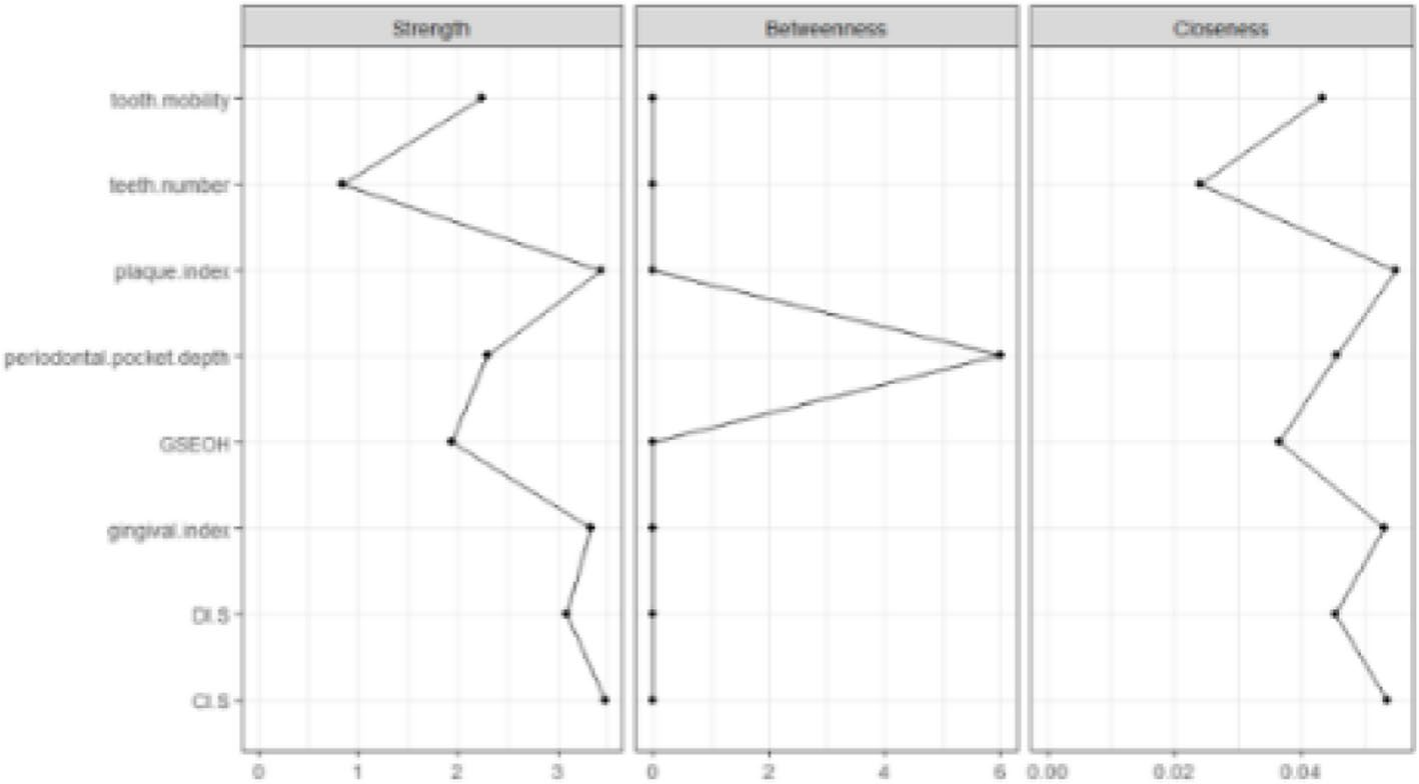
Node strength centrality estimates for oral symptoms

CI-S (r _strength_ = 3.486, r _closeness_ = 0.053), plaque index (r _strength_ = 3.442, r _closeness_ = 0.055) and gingival index (r _strength_ = 3.333, r _closeness_ = 0.053) had the largest values for strength and closeness. Teeth number had the lowest centrality (r _strength_ = 0.841, r _closeness_ = 0.024). (Fig. 2).

#### Bridge centrality

**Fig. 3:**
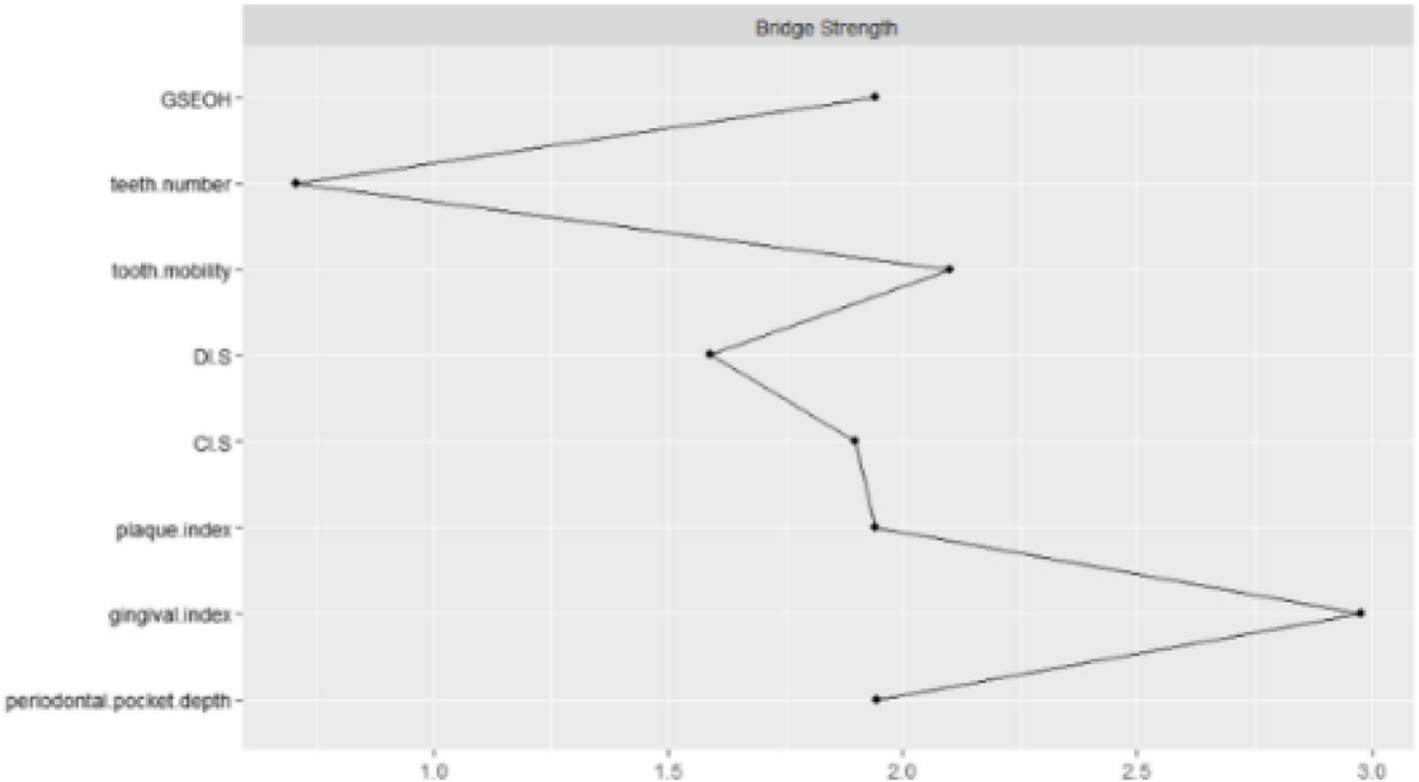
Centrality of bridge oral symptoms

Figure 3 shows the centrality of bridge symptoms of the 4 clusters. In the Periodontal health cluster, gingival index had the highest bridge strength (r _strength_ = 2.976). In the Tooth Health cluster, tooth mobility had the highest bridge strength (r _strength_ = 2.101). In the Oral hygiene index cluster, plaque index had the highest bridge strength (r _strength_ = 1.942).

#### Difference test

**Fig. 4:**
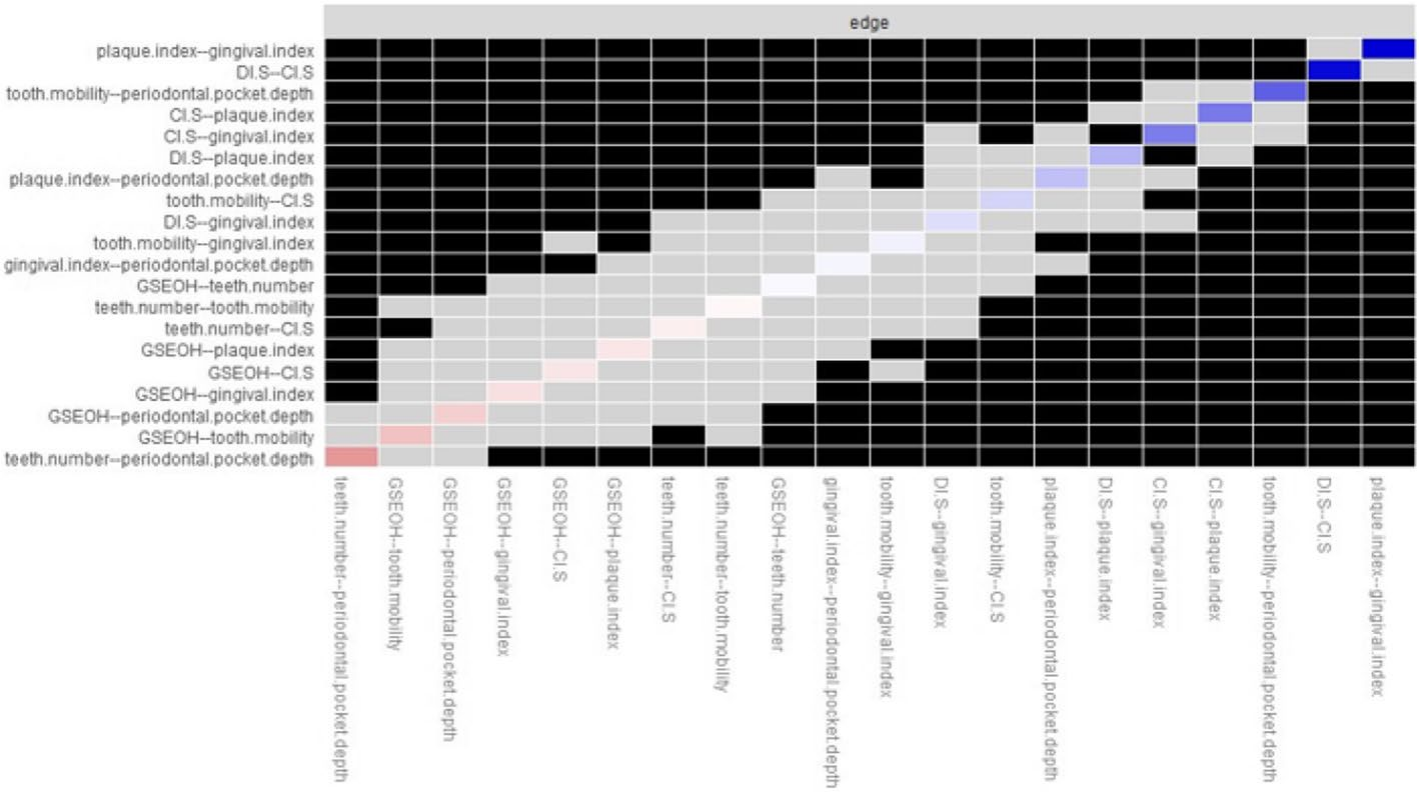
Estimation of edge weight difference by bootstrapped deference test

**Fig. 5:**
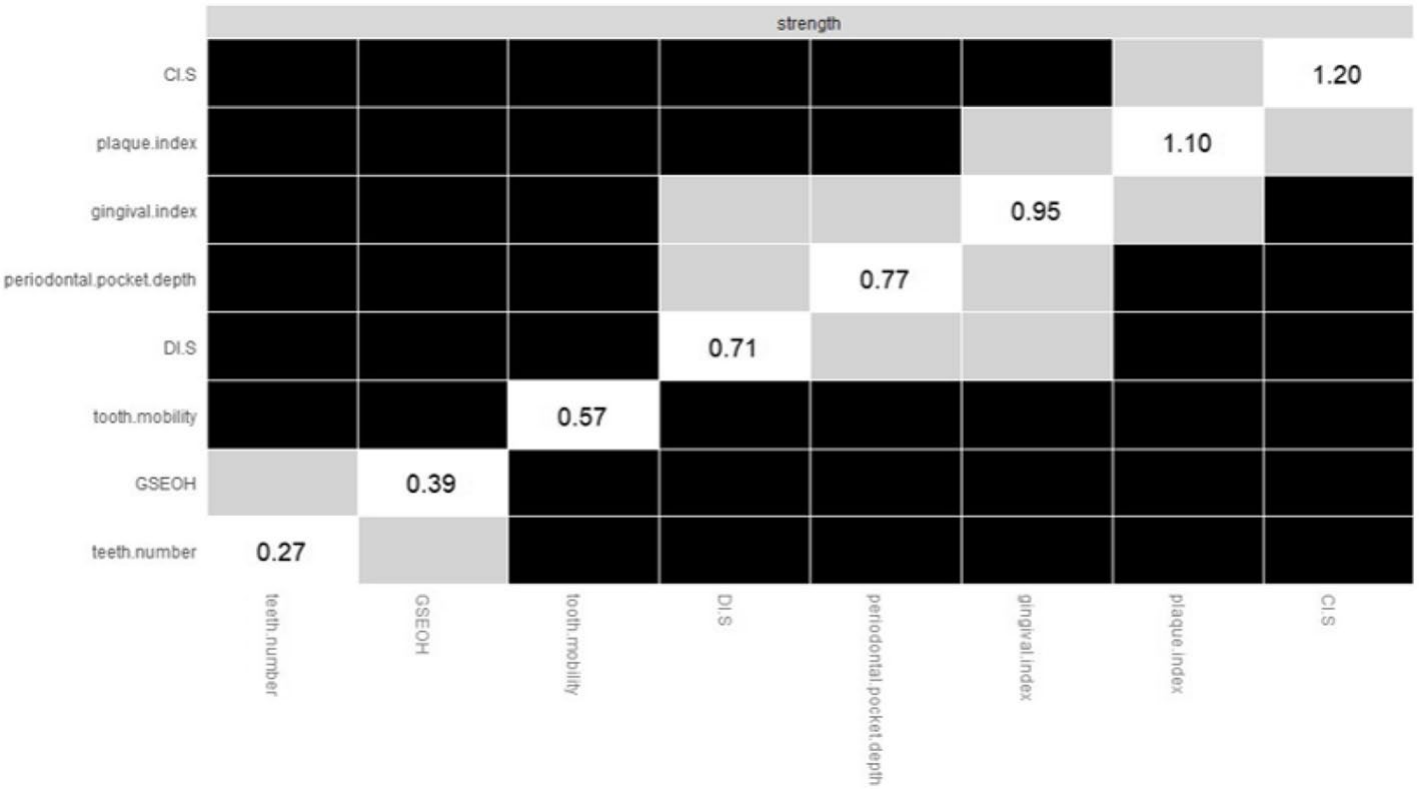
Estimation of node strength difference by bootstrapped difference test

The strongest edge weights are plaque index –gingival index, and DI-S – CI-S, and they were significantly different from the other edge weights (Fig. 4). The bootstrapped node difference test showed that CI-S significantly differed from other nodes (DTs = 1.20) (Fig. 5).

### Accuracy, and stability of symptom networks

**Fig. 6:**
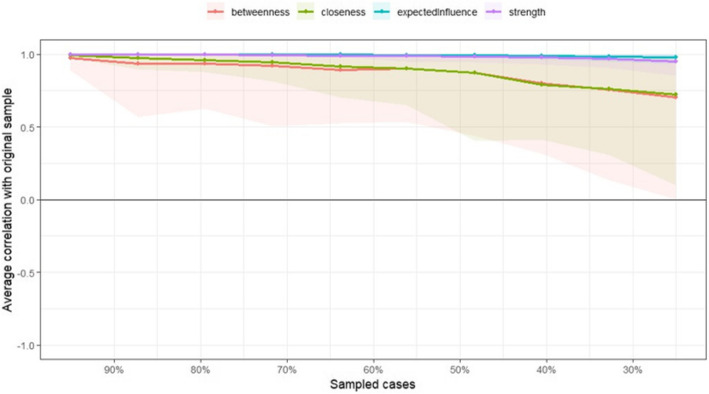
Stability of centrality indices by case dropping subset bootstrap

**Fig. 7:**
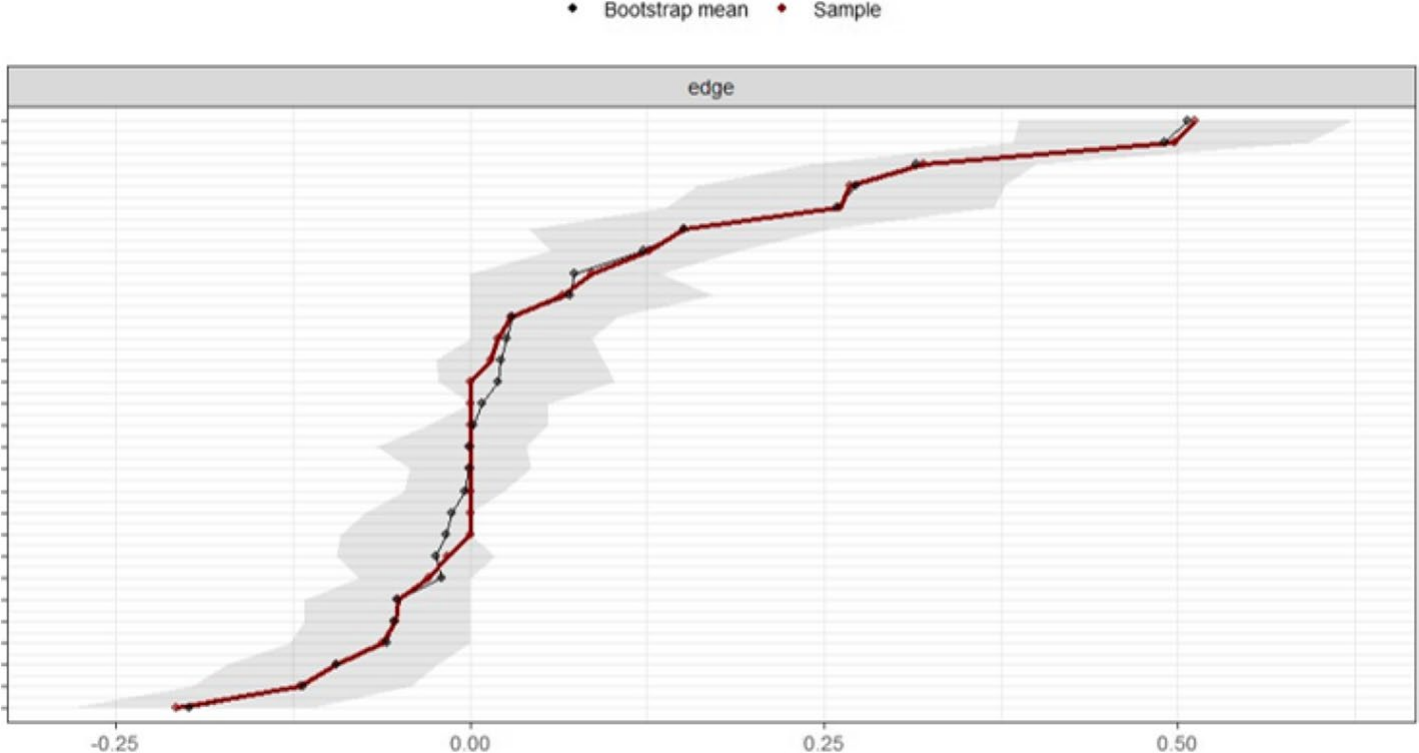
Accuracy of the edge-weights for the current network model

The correlation stability coefficient was 0.75, suggesting that the network remained stable (Fig. 6). Bootstrapped 95% CIs for the estimated edge-weights were narrow, indicating that the estimates were reliable (Fig. 7).

### Subgroup global strength invariance test

Subgroup networks were compared based on oral frailty and overall frailty. Global strength invariance tests showed no significant difference between the older adults with or without oral frailty (S = 0.027, p = 0.905), and frailty (S = 0.336, P = 0.192). (Supplementary).

Figure 1: Network of oral symptoms. Nodes represent oral hygiene symptoms, periodontal symptoms, and tooth symptoms, while edges represent partial correlations between symptoms. Edge thickness and darkness indicate the strength of these associations (with minimum and maximum edge values standardized across networks). Node colors differentiate symptom categories: deep red for periodontal symptoms, light red for hygiene symptoms, yellow for tooth symptoms, and blue for GSEOH. Edge colors denote correlation valence: green for positive and red for negative. The thick green edges between gingival index, plaque index, DI.S, and CI.S indicate strong positive correlations. In contrast, tooth symptoms (yellow nodes) and GSEOH (blue node) are positioned on the periphery of the network, suggesting their relatively minor influence on the overall network. Red edges show that GSEOH is negatively correlated with all symptoms except tooth number, while tooth number is negatively correlated only with periodontal pocket depth.

Figure 4 Bootstrapped difference test between edge weights in the network. Gray boxes indicate edges that do not significantly differ from one another. Black boxes represent edges with a significant difference from one another (α = 0.05). Blue boxes in the edge-weight plot indicate positive correlations, and orange boxes in the edge-weight plot indicate negative correlations.

Figure 5 Bootstrapped difference test between node strength of factors. Gray boxes indicate nodes that do not significantly differ from one another. Black boxes represent nodes that significantly differ from one another (α = 0.05). White boxes show the values of node strength.

Figure 6 Stability of centrality indices by case dropping subset bootstrap. The x-axis shows the percentage of cases removed at each step, while the y-axis represents the average correlation between centrality indices from the original and re-estimated networks as cases are progressively removed. The shaded regions indicate the 95% confidence intervals (CIs). The figure showed that the purple line (strength) and blue line (expected influence) maintain high correlations and narrow CIs as the sample size decreases, indicating strong stability.

Figure 7 Accuracy of edge-weights for the current network model. The red line marks the sample value, the gray area represents the CI, and the black line shows the mean edge weight estimated via bootstrap. The x-axis denotes the estimated edge weight coefficient, and the y-axis ranks edge weights from highest to lowest. The gray area is narrow, indicating high accuracy of edge weights of the current network.

## Discussion

In this study, binary logistic regression analysis indicated that teeth number and GSEOH were significantly associated with oral frailty. Interestingly, network analysis revealed that the centrality of teeth number and GSEOH was the lowest compared to other oral symptoms. Teeth number was a special and isolated symptom, and only has negative connection with periodontal pocket depth.

GSEOH was a peripheral symptom and negatively associated with all oral symptoms except for teeth number.

**Fig. 8:**
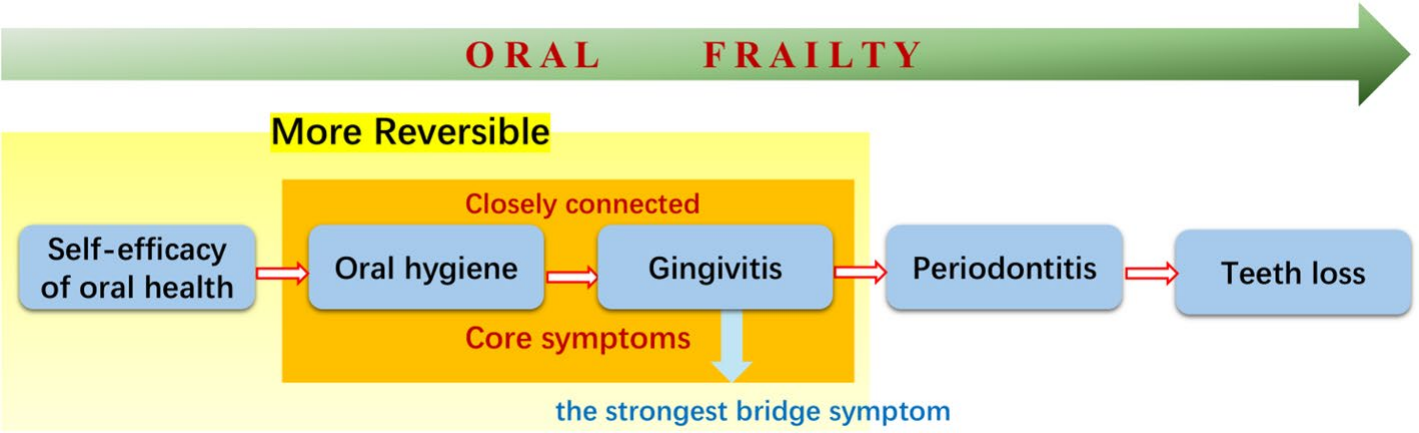
Interactions among oral symptoms

Network analysis revealed that CI-S, plaque index, and gingival index were identified as core symptoms in older adults. CI-S and plaque index, together with DI-S, are used to assess oral hygiene status. The strong edge thickness observed between oral hygiene status and gingival index indicates a close and robust interaction between these factors. Although this finding differs from our initial hypothesis, it highlights the critical importance of oral hygiene status and its association with oral diseases, offering valuable insights for clinical interventions. Moreover, network analysis revealed that the gingival index was the strongest bridge node, suggesting that maintaining and improving gingival health could play a pivotal role in preventing the further progression of oral frailty. (Fig. 8).

In the network of the general and oral frailty population, teeth number was negatively correlated only with periodontal pocket depth, while in population without oral frailty, teeth number was related with more symptom nodes. However, it has been proven that periodontitis is a major cause of tooth loss [28]. The paradoxical phenomenon observed could be due to the study examining functional teeth rather than just natural teeth. Functional teeth include both natural teeth and prosthetic teeth. The significant role of periodontal pocket depth mentioned could also support this hypothesis. Periodontal pocket depth is an important indicator of periodontal diseases, and patients with severe periodontal are more likely to experience implants loss and related complications [29, 30]. In the study, teeth number is proved to be significantly associated with oral frailty, consistent with preview research [6]. Three main pathways might explain the association. The first pathway involves periodontal disease-related tooth mobility, loss, and decreased occlusal support, which can impair masticatory function [6, 31, 32]. The second concerns damaged masticatory function caused by advanced periodontal disease. The two pathways could reduce masticatory efficiency, compromise digestion and nutrient absorption, thus lead to insufficient nutrition [33]. The third pathway could impact social relationships because of periodontitis-related halitosis and dental aesthetics, increasing the likelihood of depression in older adults [3, 6]. Prosthodontic treatment after tooth loss can block the first and third pathways. Therefore, it is recommended that communities adopt multiple strategies to increase the denture restoration rate among older residents, such as publicly funding or subsidizing the cost of dental care, as well as conducting domiciliary dental care and mobile dental clinic visits [34, 35]. Additionally, communities should implement periodontal maintenance programs to prolong the survival of natural teeth [36].

The regression analysis indicated a significant correlation between self-efficacy of oral health and oral frailty. Symptom network analysis revealed that self-efficacy of oral health was associated with all oral symptoms except for teeth number. Studies have demonstrated the close relationship between self-efficacy and oral health [37, 38]. The critical relationship may be attributed to the fact that self-efficacy is a predictor of health behavior changes, and dental diseases are behaviorally mediated. Thus, appropriate modifications in oral health behaviors can influence the trajectory of dental disease progression [19, 39]. Therefore, it’s recommended to implement oral health promotion programs aimed at enhancing older adults' attitudes and self-efficacy regarding oral health. Such programs can foster positive oral health behaviors and ultimately improve oral health status [40].

The results suggest that oral hygiene status and gingivitis are the most central and closely connected symptoms within the symptom network. Among these, gingivitis serves as the bridge symptom. Oral hygiene status has a deep impact on overall oral health. The oral cavity accommodates a diverse array of microorganisms [41]. The transition process from periodontal health to advanced stages of periodontitis is associated with a microbial shift from symbionts to dysbiosis [42]. Microbial dysbiosis form pathogenic biofilm over the periodontal surface and gingival region, visible as plaque [43]. plaque accumulation and poor oral hygiene have been reported as crucial risk factors for periodontitis [44]. Poor oral hygiene increases the risk of periodontitis by 2 to 5 times [45]. The result and previous studies both suggested gingivitis is a potential symptom between poor oral hygiene and periodontitis [46, 47]. It is encouraging that gingivitis can be reversed with improved oral hygiene, preventing its progression into irreversible periodontitis that damages tooth attachment and supporting bone [46, 48]. Therefore, maintaining oral hygiene is crucial and can be achieved through biofilm control via educational interventions, tailored oral health programs, professional tooth cleaning, and chemical biofilm management using chlorhexidine, etc. [49].

Family income and overall frailty are also significantly associated to oral frailty. Higher-income individuals are less likely to experience oral frailty, consistent with previous research findings. They tend to be more aware of oral health, adhere to regular oral hygiene practices such as frequent cleanings and checkups, receive high-quality dental care, and purchase high-quality oral hygiene products [5, 50]. The results demonstrated that oral frailty is closely associated with overall frailty, consistent with previous studies showing a strong link between the two. Furthermore, oral frailty has been identified as a predictor of overall frailty [4, 51–53]. Three pathways may explain this relationship. The first pathway is oral function decline: Poor oral function, a key characteristic of oral frailty, contributes significantly to overall frailty. A systematic review has shown that fewer teeth and reduced masticatory function are longitudinally associated with the development of frailty [54]. The second pathway is social frailty: social frailty is directly associated with both oral frailty and overall frailty, providing another explanation for the strong link. Declines in social function are often accompanied by reduced health literacy, which can impair self-management abilities. Oral health, tied closely to lifestyle habits such as diet and hygiene, may deteriorate due to insufficient management, further contributing to oral frailty [55, 56]. Additionally, social frailty may lead to fewer speaking opportunities, reducing tongue movements and subsequently lowering tongue pressure [57]. Meanwhile, social frailty is also linked to decreased physical activity, a known risk factor for overall frailty [58]. The third pathway is malnutrition: Oral frailty often results in significant challenges with chewing and swallowing, leading to decreased nutritional intake. It has been well-established that malnutrition is associated with overall frailty [59].

Moreover, overall frailty can also negatively impact on oral health. Watanabe et al. found that frail older adults exhibit significantly reduced occlusal force, masseter muscle thickness, and oral diadochokinesis (ODK) rates compared to pre-frail and non-frail individuals [60]. One possible explanation is that frail older adults are at a higher risk of polypharmacy due to the need to manage multiple comorbidities [61]. The use of more than three oral medications per day is associated with dry mouth, which increases the risk of oral function impairments such as candidiasis, dental caries, and oropharyngeal dysphagia [62]. This highlights the close and complex interaction between oral health and frailty, forming a mutually reinforcing vicious cycle [63]. Notably, most oral health conditions are reversible. Preventing oral disease and restoring oral function could play a crucial role in preventing frailty or delaying its onset [60, 64]. Furthermore, this study revealed that the strength and bridge strength of periodontal pocket depth in frail populations are higher compared to overall and non-frail populations. Interestingly, this is not the case in the subgroup analysis of oral frailty. This suggests that future research should further analyze the causal relationships between oral symptoms, particularly periodontal pocket depth, oral frailty, and overall frailty.

## Conclusion

This is the first study that explored the relationship of oral symptoms and oral frailty using network analysis. The result proves that functional teeth number and GSEOH play significant roles in oral frailty, and oral hygiene status and gingivitis are the most central and closely connected symptoms. Future research should further investigate the causal relationships between oral symptoms, particularly periodontal pocket depth, oral frailty, and overall frailty. The study recommends that communities implement routine oral functional assessments to increase the denture restoration rate and identify oral frailty at an early stage, as well as educational and promotional programs aimed at maintaining oral hygiene and function. Additionally, the study emphasizes improving the accessibility of dental services in communities through government funding and home-based services.

The limitations of this study are as follows. Firstly, the study employed a cross-sectional design, which limits the ability to establish causal relationships between influencing factors and oral frailty. Future studies should consider adopting a longitudinal design to better understand the temporal relationships and causality among these factors. Secondly, the study utilized convenience sampling, which may have introduced selection bias and limited the representativeness of the sample. Future research is encouraged to employ random sampling methods to enhance the external validity and generalizability of the findings. Thirdly, the study did not distinguish the statuses of different types of prosthetic teeth, such as denture teeth, dental bridges, or implants. Future research should conduct a detailed classification of prosthetic teeth to explore their specific associations with oral frailty.

## Supplementary Information

Supplementary Material 1.

## Data Availability

Data are available on reasonable request. All data relevant to the study are included in the article. The data related to this study are available from the corresponding author on reasonable request and pending additional ethical approval.
