# Supplementary material for: The symptom network of oral health conditions in older populations with oral frailty: a cross-sectional study
Source: BMC Oral Health. 2025 Apr 1;25:471. doi: 10.1186/s12903-025-05795-9 (PMC11963664; doi:10.1186/s12903-025-05795-9)
Supplement: Supplementary file 1 — Supplementary Material 1. [file 12903_2025_5795_MOESM1_ESM.docx]

1. **Comparison of networks in older population with and without oral frailty**

**1.1 Networks of oral symptoms**
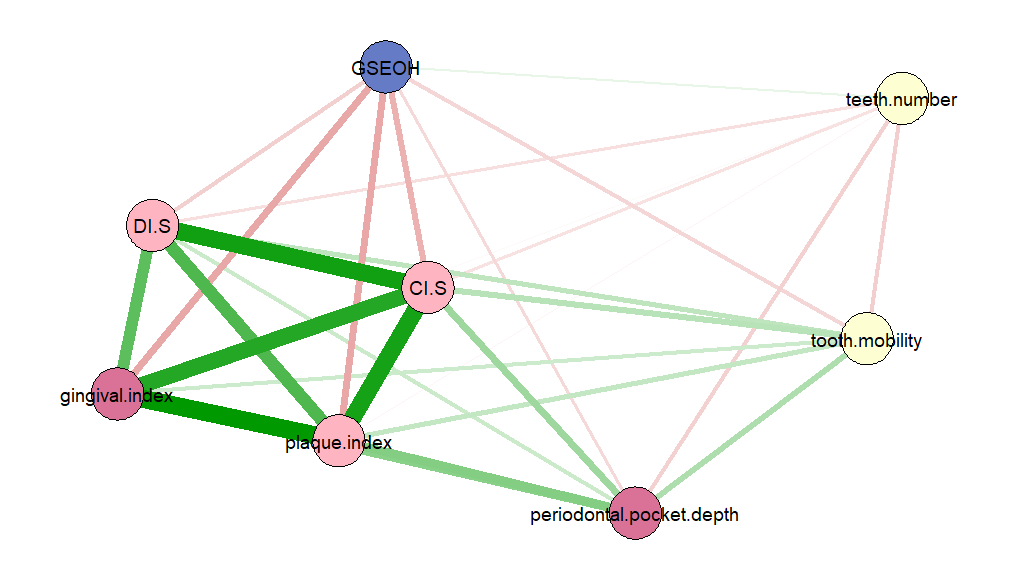


**
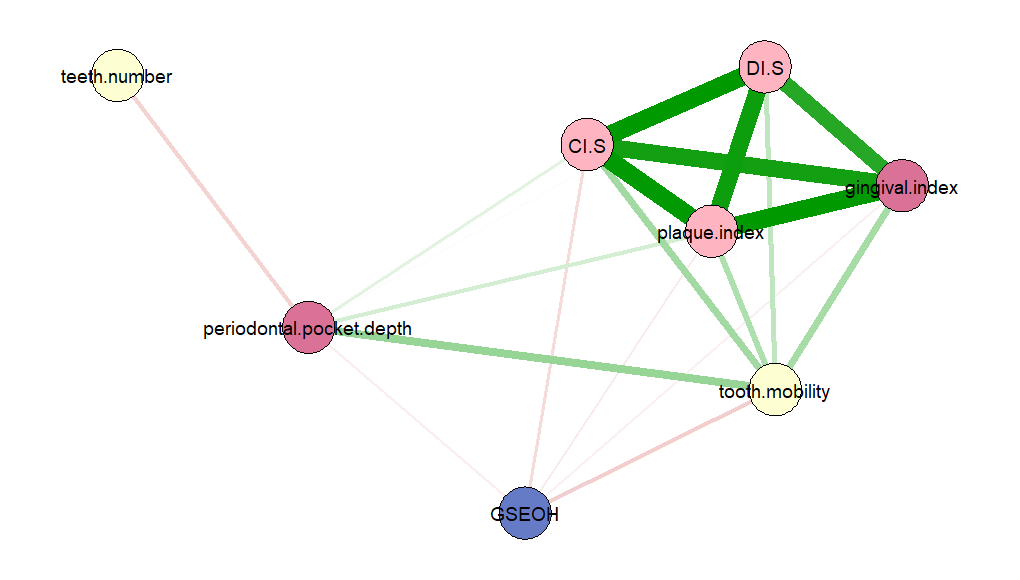
 population without oral frailty**

**population with oral frailty**

**
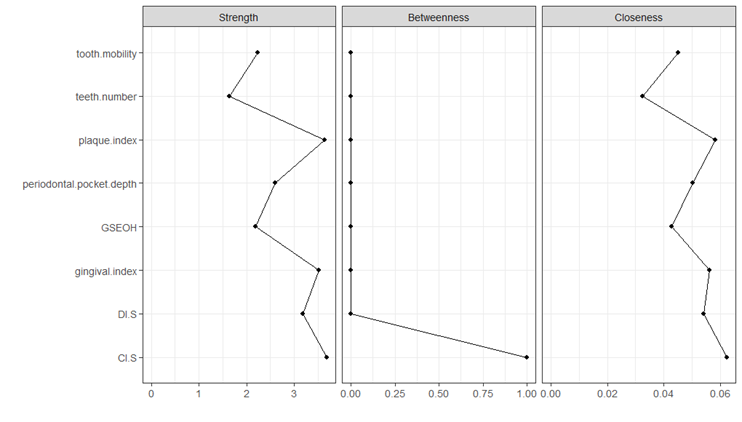
1.2 Node strength centrality estimates for oral symptoms**

**population without oral frailty**

**
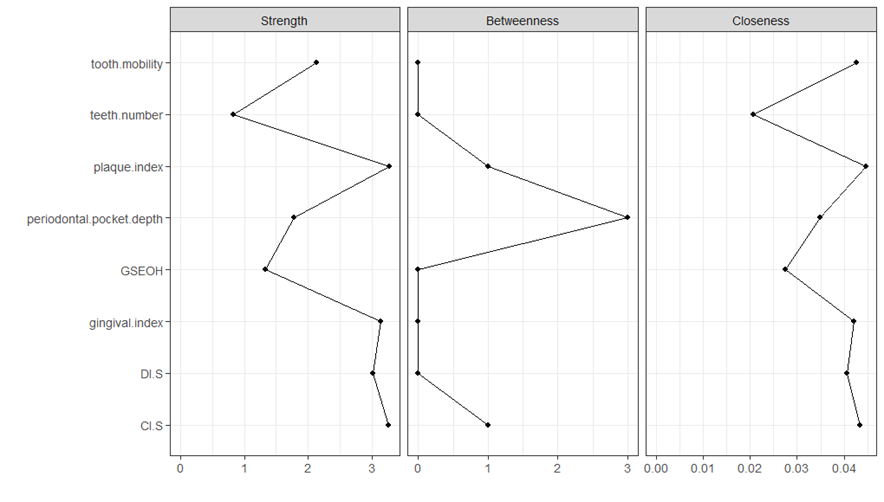
**

**population with oral frailty**

**1.3 the centrality of bridge oral symptoms**

**
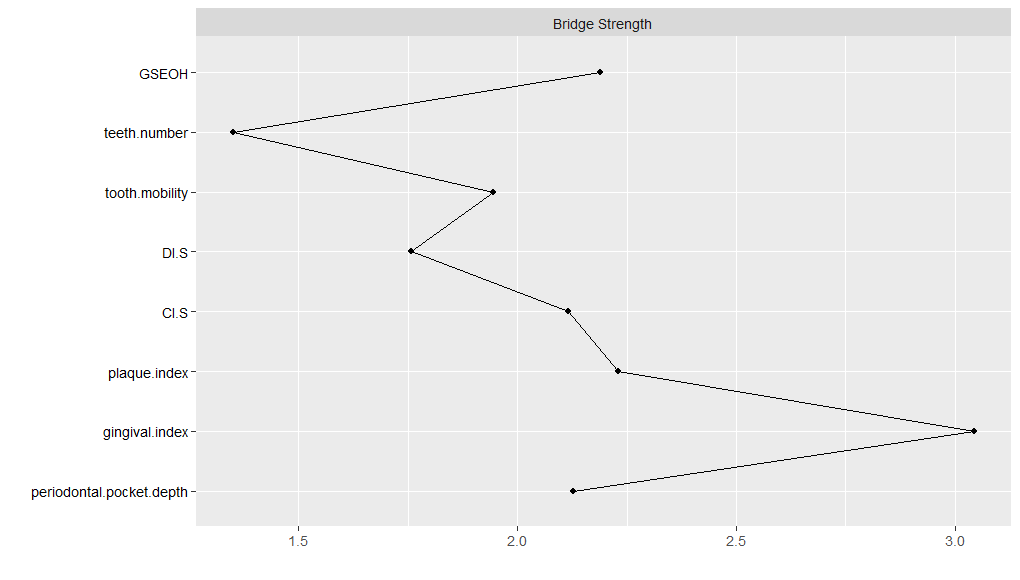
**

**population without oral frailty**

**
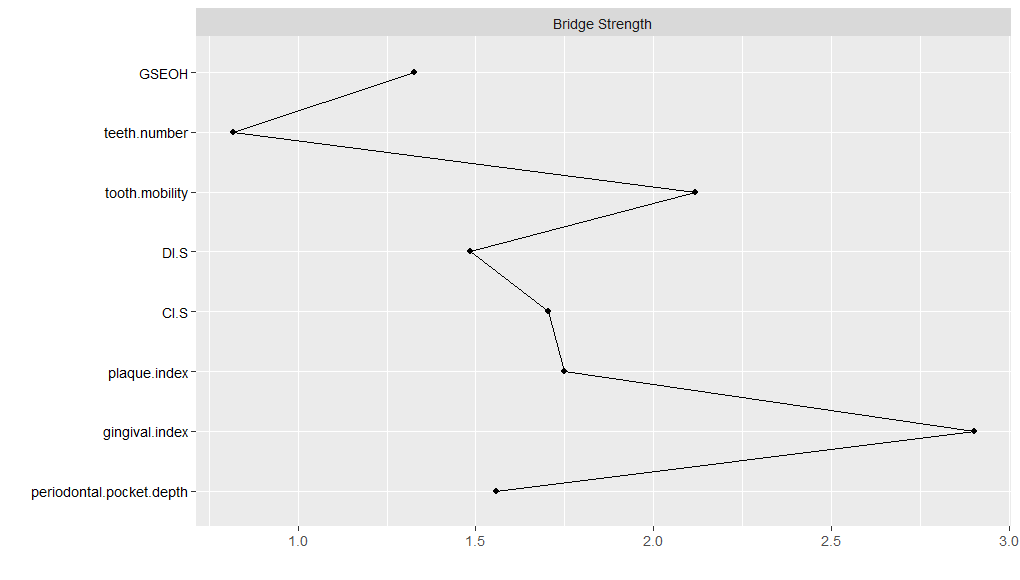
**

**population with oral frailty**

**
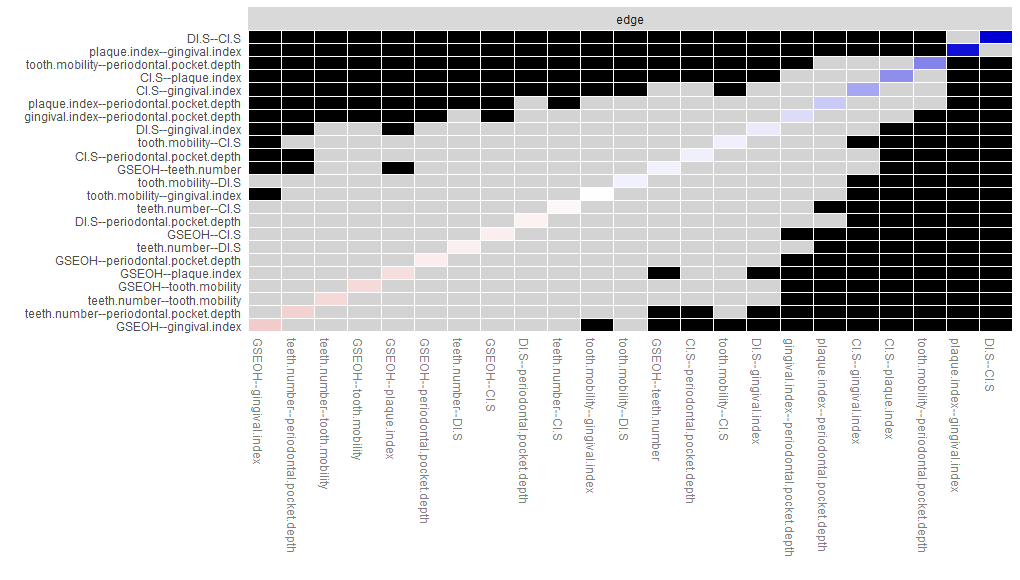
1.4 Estimation of edge weight difference by bootstrapped difference test**

**population without oral frailty**

**
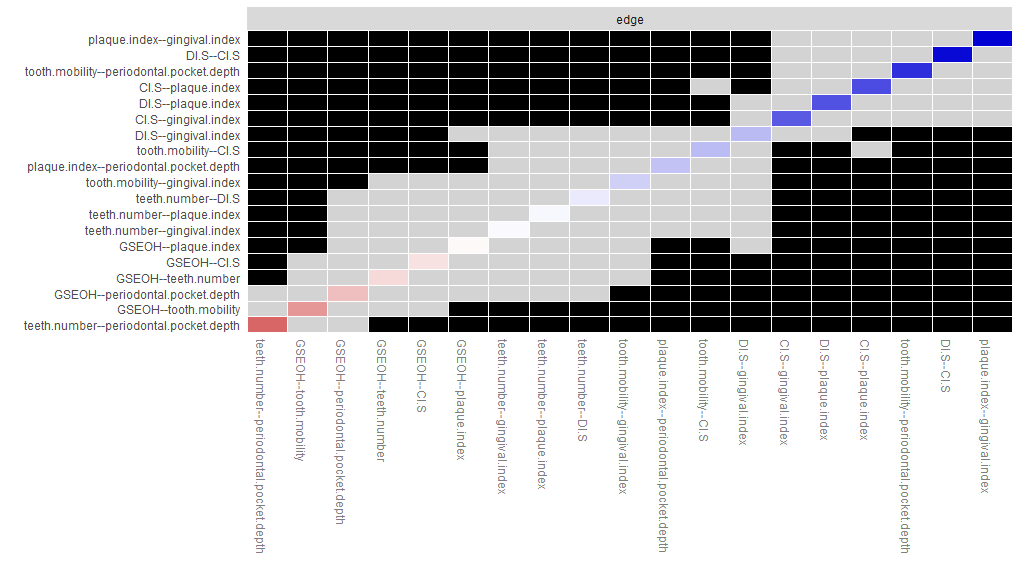
**

**Figure S-5 Estimation of node strength difference by bootstrapped difference test**

**population with oral frailty**

**1.5 Stability of centrality indices by case dropping subset bootstrap**

**
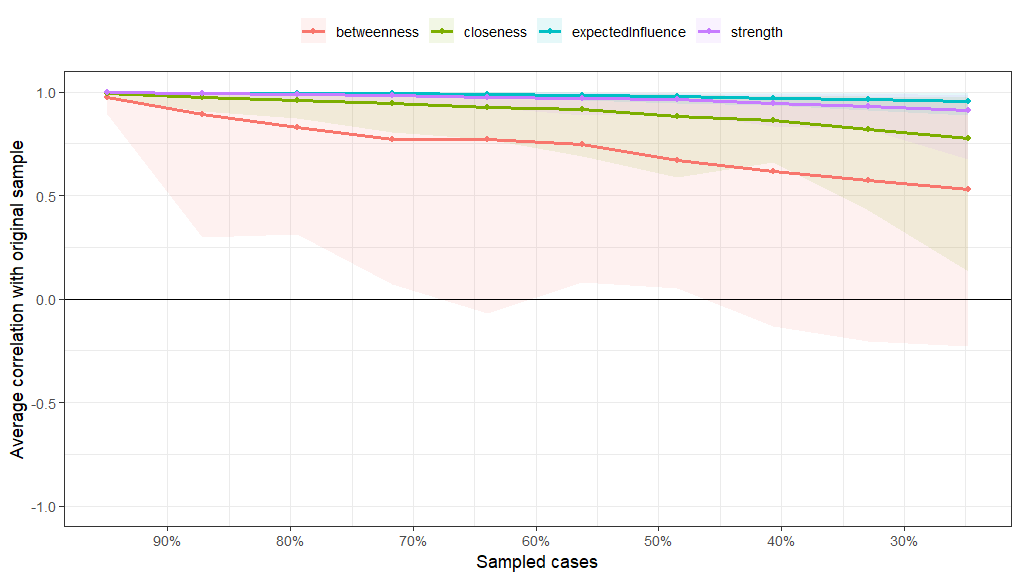
**

**population without oral frailty**

**
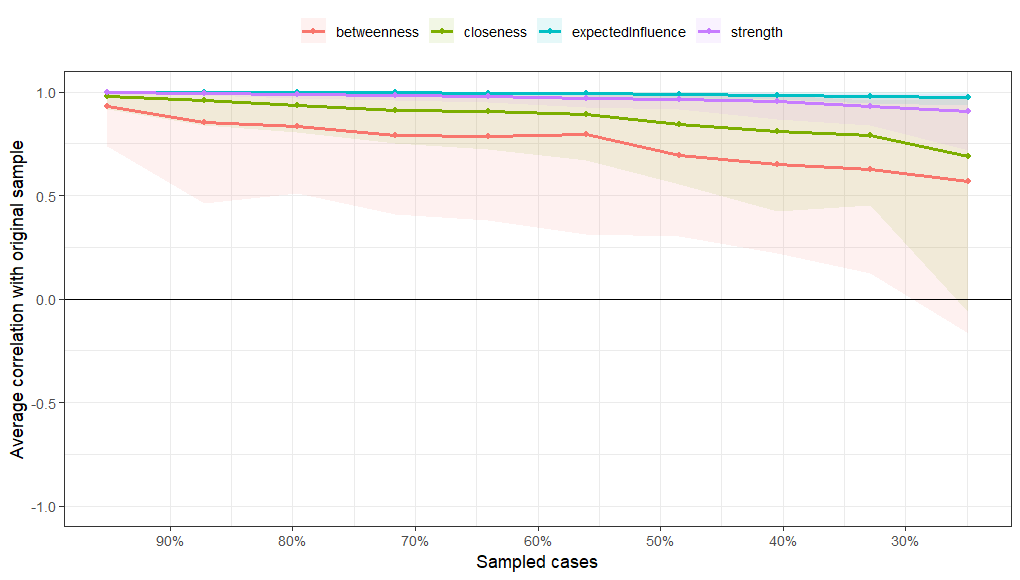
**

**population with oral frailty**

**1.6 global strength test result**

**
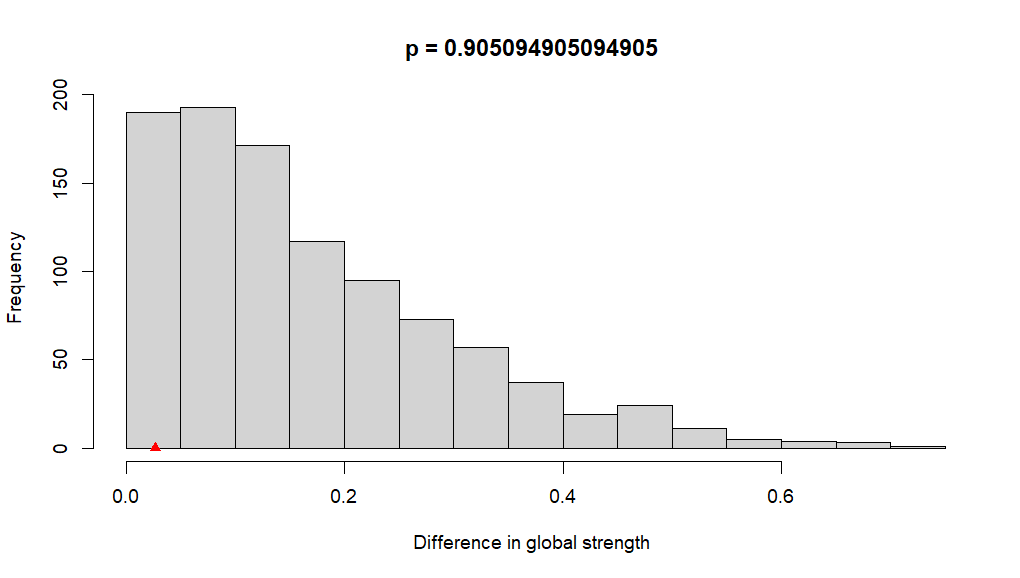
**

A plot of bootstrap value of the difference in network global strength. The difference was not significant (network strength among older adults without oral frailty: 3.030; among older adults with oral frailty : 3.060; S: 0.027, p=0.905).

**1.7 network test results**


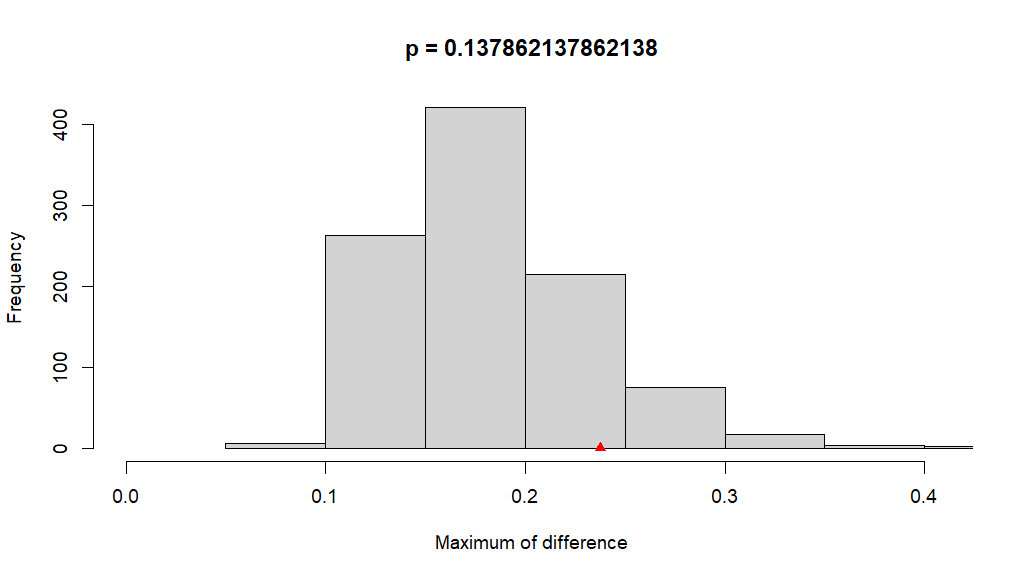


A plot of bootstrap value of the maximum difference in any of the edge weights. The difference was not significant (M=0.238, p=0.138)

1. **Comparison of networks in older population with and without frailty**

**2.1 Networks of oral symptoms**


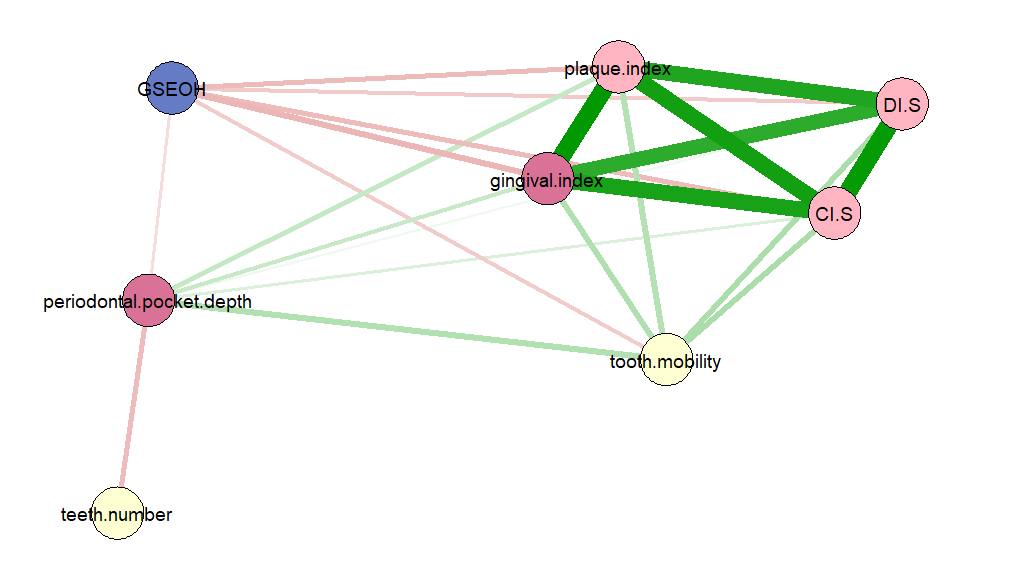


**population without frailty**


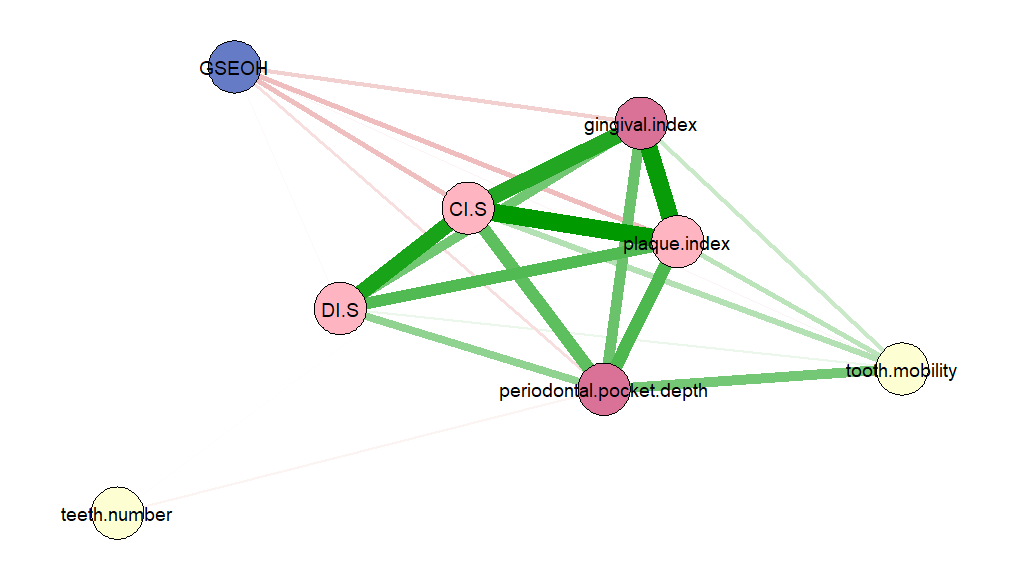


**population with frailty**

**2.2 Node strength centrality estimates for oral symptoms**


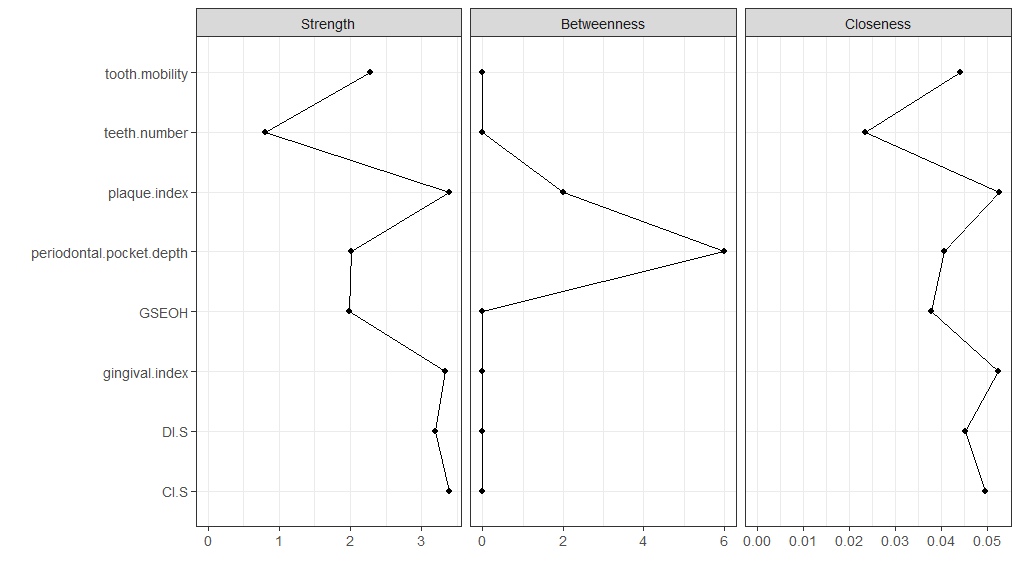


**population without frailty**


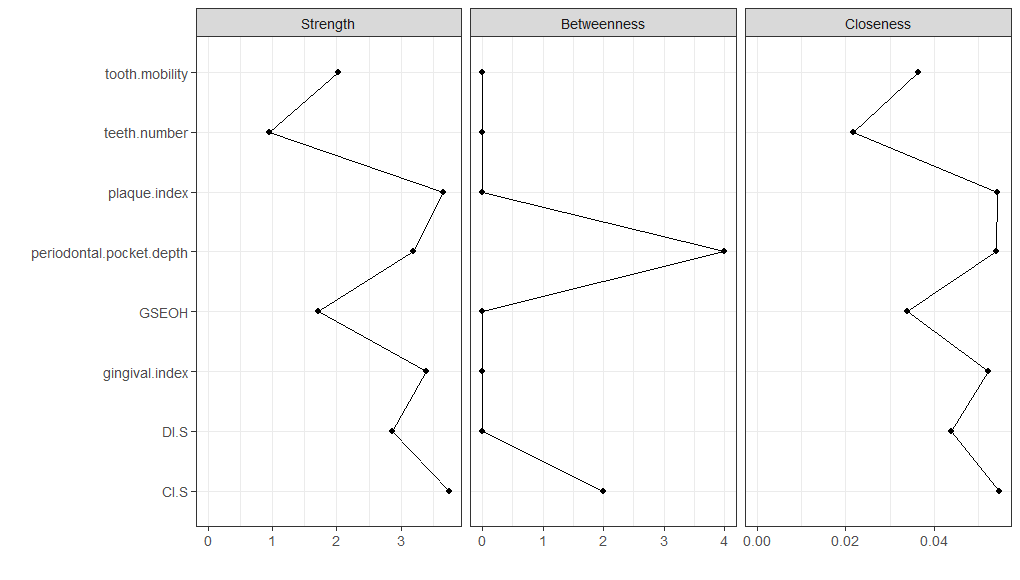


**population with frailty**

**2.3 the centrality of bridge oral symptoms**


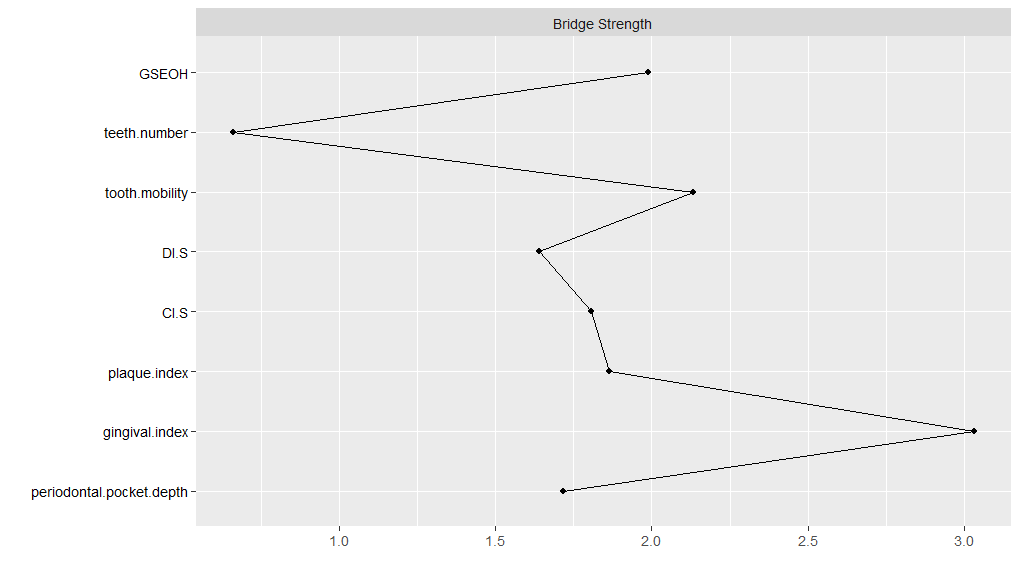


**population without frailty**


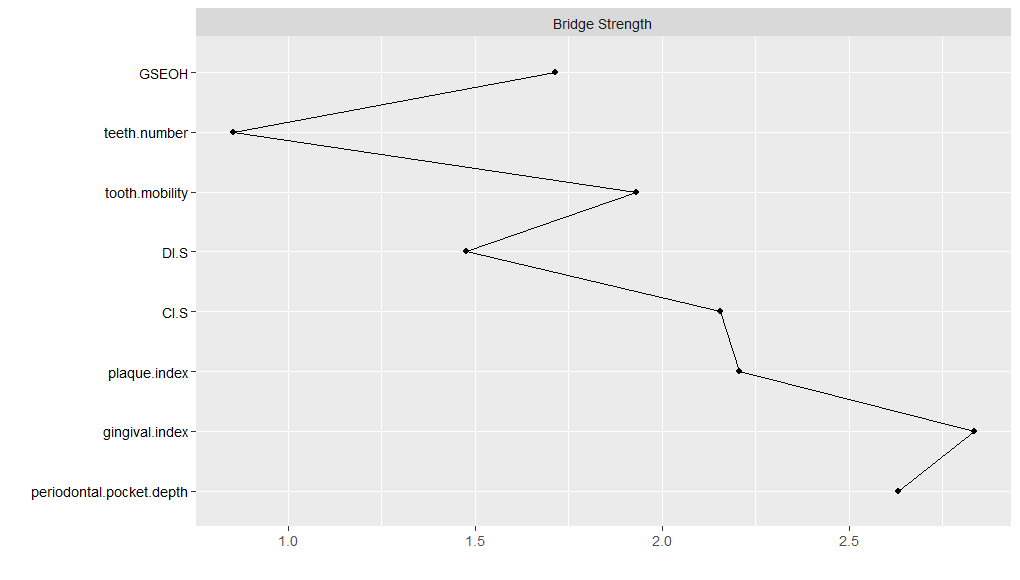


**population with frailty**

**2.4 Estimation of edge weight difference by bootstrapped difference test**

**population without frailty**


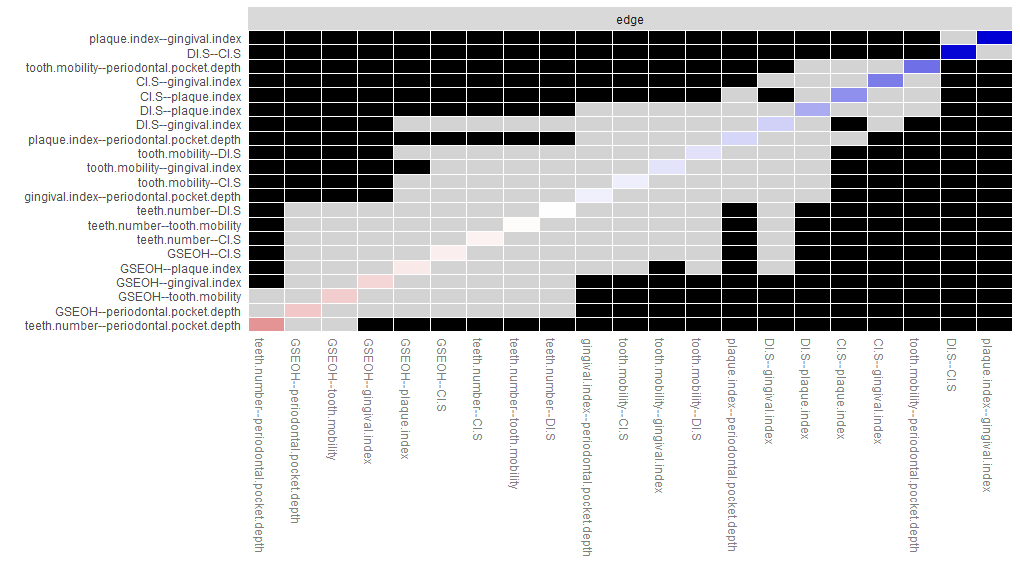


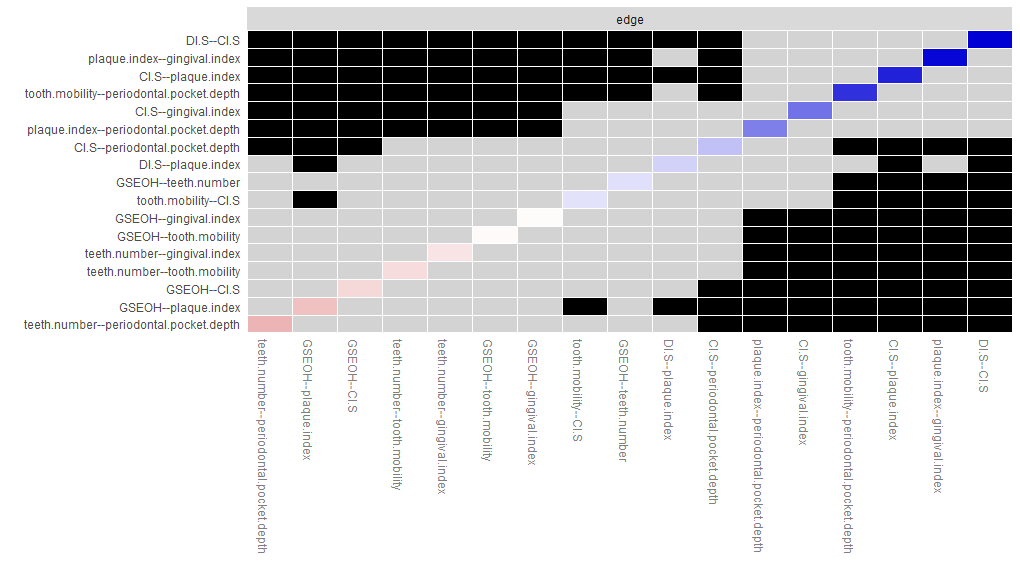


**population with frailty**

**2.5 Stability of centrality indices by case dropping subset bootstrap**

**population without frailty**


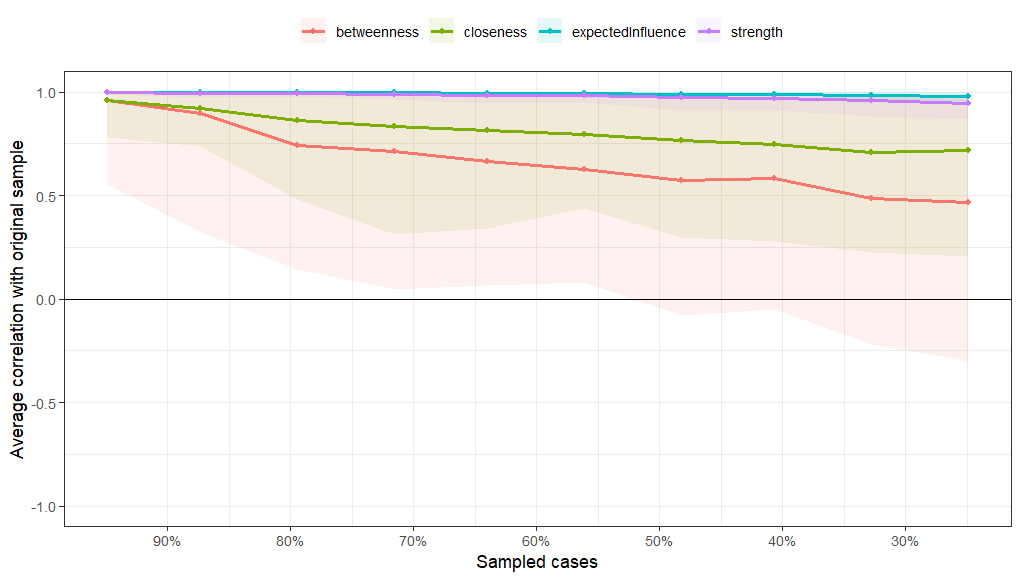


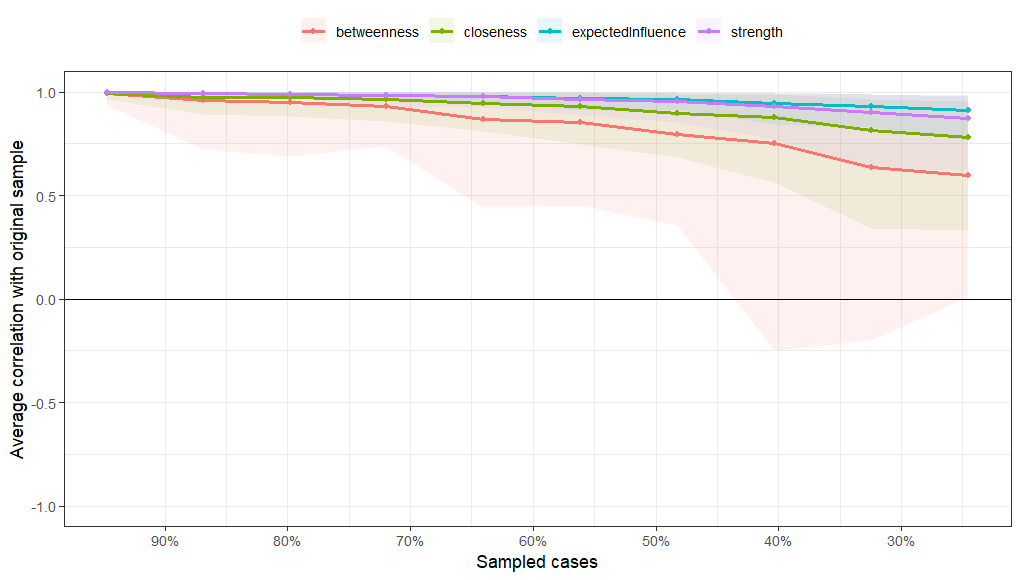


**population with frailty**

**2.6 network test results**


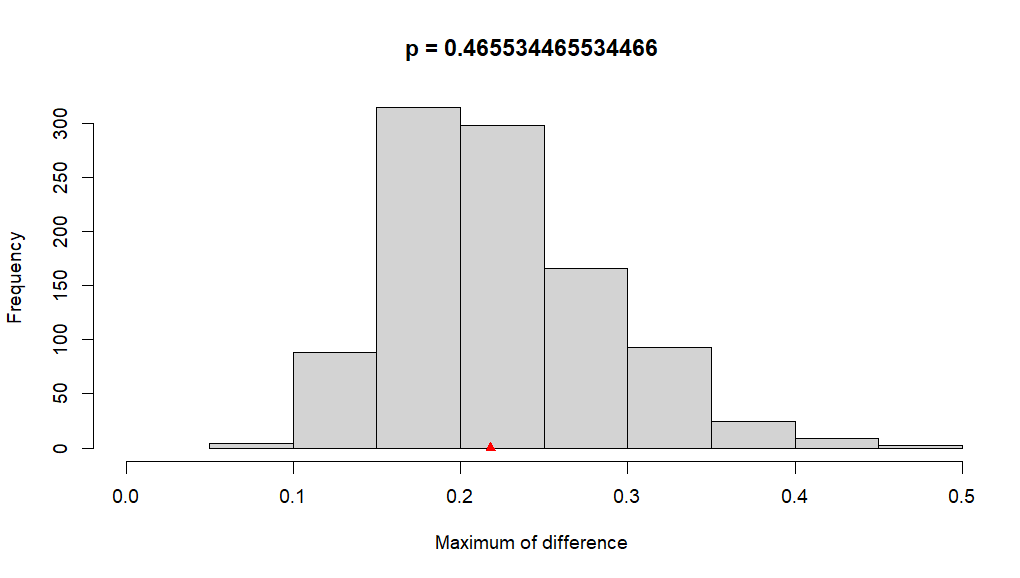


A plot of bootstrap value of the maximum difference in any of the edge weights. The difference was not significant (M=0.219, p=0.466)

2.7 global strength test result

A plot of bootstrap value of the difference in network global strength. The difference was not significant (network strength among older adults without frailty: 3.119; among older adults with frailty: 2.783; S: 0.336, p=0.192).
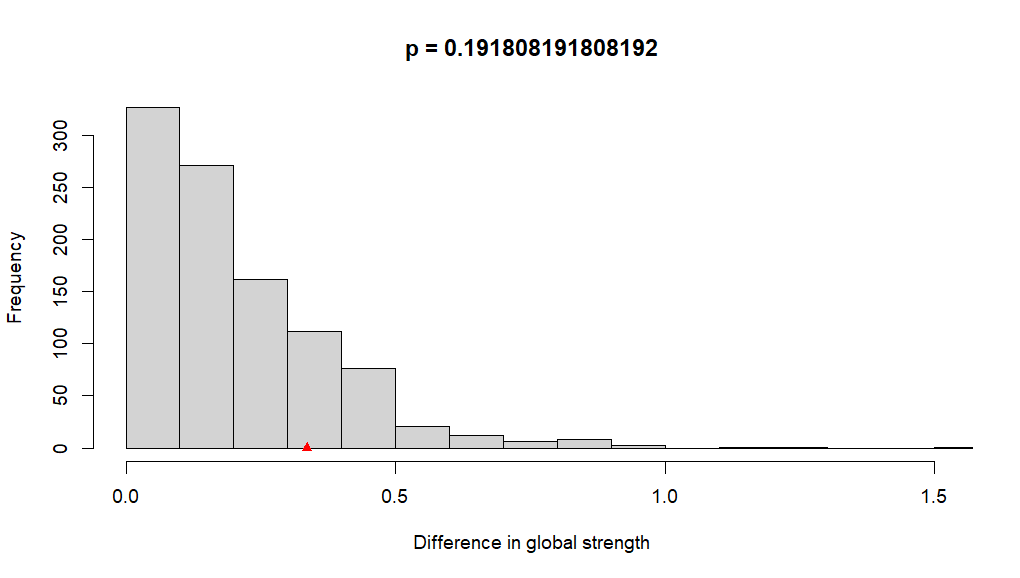


1. **Variable coding**

**3.1 Variable Coding in Table 3**

| **Parament** | **Reference Group** | **Coding Method** |
| --- | --- | --- |
| Gender | Male | 1 = Female |
| Education | / | 1 = Junior high and below 2 = High school/secondary school 3 = College/university and above |
| Family Income | / | 1 = <20k 2 = 20k~50k 3 = 50k~80k 4 = >80k |
| Tooth Mobility | / | 1 = <1 mm mobility 2 = >1 mm but not depressible 3 = >1 mm and depressible |
| DI-S | / | 1 = No debris or stain present 2 = Soft debris or extrinsic stains covering ≤1/3 of tooth surface 3 = Soft debris covering >1/3 but ≤2/3 of the tooth surface 4 = Soft debris covering >2/3 of the tooth surface |
| CI-S | / | 1 = No calculus present 2 = Supragingival calculus covering ≤1/3 of the tooth surface 3 = Supragingival calculus covering >1/3 but ≤2/3 of the tooth surface, or subgingival calculus flecks around the cervical portion 4 = Supragingival calculus covering >2/3 of the tooth surface, or continuous heavy band of subgingival calculus around the cervical portion |
| Plaque Index | / | 1 = No visible plaque 2 = Plaque islands on the proximal surfaces, 3 = Plaque islands cervical to the bracket in addition to the proximal surfaces 4 = Plaque covering >1/3 of the surface cervical to the bracket |
| Gingival Index | / | 1 = Healthy gums 2 = Slight color changes, mild edema, no bleeding on probing 3 = Edema with slight redness and bleeding on probing 4 = Severe edema, redness, ulceration, and spontaneous bleeding |
| Periodontal Pocket Depth | / | 1 = ≤3mm 2 = 4～5mm 3 = ≥6mm |
